# Functionality and Storability of Cookies Fortified at the Industrial Scale with up to 75% of Apple Pomace Flour Produced by Dehydration

**DOI:** 10.3390/foods8110561

**Published:** 2019-11-08

**Authors:** Snežana Zlatanović, Ana Kalušević, Darko Micić, Jovanka Laličić-Petronijević, Nikola Tomić, Sanja Ostojić, Stanislava Gorjanović

**Affiliations:** 1 Institute of General and Physical Chemistry, P.O. Box 45, 11158 Belgrade 118, Serbia; micic83@gmail.com (D.M.); ostojicsanja404@gmail.com (S.O.); 2 Institute of Meat Hygiene and Technology, Kaćanskog 13, 11000 Belgrade, Serbia; anakalusevic@gmail.com; 3 Faculty of Agriculture, University of Belgrade, P.O. Box 14, 11080 Belgrade, Serbia; jovankal@agrif.bg.ac.rs (J.L.-P.); tsnikola@agrif.bg.ac.rs (N.T.)

**Keywords:** antioxidant, apple pomace, dietary fiber, cookies, gluten free, polyphenolics

## Abstract

Apple pomace flour (APF) with high content of dietary fibers (DF), total polyphenolics (TPCs) and flavonoids (TFCs) was produced at the industrial scale. Bulk and tapped density, swelling, water and oil holding capacity, solubility and hydration density of fine and coarse APF with average particle size 0.16 and 0.50 mm were compared. The effect of wheat flour substitution with 25%, 50% and 75% of fine and coarse APF was studied upon cookies production at the industrial scale and after one year of storage. Coarse APF performed better in respect to sensorial properties, content and retention of dietary compounds and antioxidant (AO) activity. The cookies with optimal share of coarse APF (50%) contained 21 g/100 g of DF and several times higher TPC, TFC as well as AO activity than control cookies, retained well health promoting compounds and maintained an intensely fruity aroma and crispy texture. They were acceptable for consumers according to the hedonic test.

## 1. Introduction

Apple (*Malus* sp.) is the most processed fruit that generates a high amount of apple pomace (AP) composed of peel, pulp, stems and seeds. The composition, health benefits and safety of AP were analyzed comprehensively [1]. It is high in dietary fibers (DF; soluble pectins, β-glucans, galactomanan gums and insoluble lignin, cellulose and hemicelluloses) [2,3] with antioxidant (AO), antitumor and hepatoprotective activities [4,5]. AP also represents a rich source of polyphenolic compounds (catechins, procyanidins, phloridzin, phloretin glycosides, caffeic and chlorogenic acid, quercetin and cyanidin glycosides) with antioxidative, antiproliferative, anti-inflammatory, antidiabetic and cardioprotective effects [6,7,8,9]. AP’s huge potential for conversion into edible products with additional nutritional value and flavor was demonstrated [10] but not fully exploited. Commercially available food items with declared AP in their composition are still rare.

To obtain a non-perishable food ingredient, the high water content in AP (approx. 75%) needs to be reduced. The removal of water without affecting nutritional and functional properties represents a major obstacle to commercial use of AP. Various drying techniques to reduce moisture in AP have been tested. Among them, time- and energy-consuming freeze drying retains the highest level of bioactive compounds [11,12,13,14]. Recently, an efficient low-cost drying method was developed to dehydrate AP at industrial scale. The time required to reduce moisture, as well as energy consumption, was much lower than in freeze drying [15]. Dehydrated AP was ground and apple pomace flour (APF), with high DF, polyphenolics and flavonoids content, distinct antioxidant (AO) activity, antidiabetic and antilipemic effect was produced [16]. High temperature of glass transition and low activity of water indicate stability throughout the shelf life, while thermal stability up to 220 °C confirms APF applicability as an ingredient in bakery and confectionery products [17].

Due to the increasing incidence of various metabolic disorders, obesity and gluten intolerance, there is an interest in developing fortified products with a high content of DF and AOs, reduced content of gluten and pleasant flavor. As ready-to-eat, quite durable products with long shelf life, cookies are very popular snacks. In addition, they represent a matrix suitable for fortification, thus providing an opportunity for the intake of important nutrients. While there are many studies of AP-enriched cookies, they still are not standard products. Most studies examining the effect of AP supplementation on the quality of biscuits were conducted at the laboratory level, using AP produced and/or dried in laboratory conditions. The majority of studies also reported supplementation, which does not exceed 30% of ordinary flour [2,10,18,19]. Based on sensory evaluation, cakes, bread and biscuits can be enriched with 5%, 10% and 15% of AP, respectively [20]. According to Jung et al. [21], cookies fortified with up to 20% AP had a pleasant fruit aroma and were found to be softer, chewier and damper than the control. Addition of AP in brown rice flour up to 10% did not cause significant change in overall acceptability of gluten-free crackers [22]. The study conducted by Kaushal and Joshi showed that as much as 30% AP powder could be incorporated in preparation of good quality cookies [23]. According to Saeed et al. [24], all sensory parameters decreased in cookies with 40% of AP. Oatmeal cookies made with 30%, 40% and 50% of AP were characterized as moderately acceptable [25].

This study deals with apple pomace flour produced by applying recently developed dehydration technology, and cookies obtained there of APF, both produced at the industrial scale level. Technological characteristics of fine and coarse APF, and degree of fortification of cookies made with 25%, 50% and 75% of APF were determined. Optimal share and preferred granulation of APF in respect to cookies formulation was established based on the increase of DF, total polyphenolics (TPCs), flavonoids (TFCs) and AO activity and sensorial properties investigated by an experts panel employing the scoring method, as well as the preservation of both functional and sensorial properties upon a common period of storage (a year). In addition, cookies with the optimal share of APF of preferred granulation as chosen by the sensory panel were tested using the hedonic test. Generally, the main purpose of research was conducted through determination of dietary compounds content and technological properties of industrially produced APF. This study represents a step towards fortifying popular types of cookies that lack dietary fibers and AOs at the industrial scale, by replacing a significant portion of wheat flour with APF, without compromising cookies acceptability, thus providing reliable insight in fortified cookies functionality and storability.

## 2. Materials and Methods

### 2.1. Materials

#### 2.1.1. Chemicals

Gallic acid, Folin-Ciocalteu’s reagent, hydrochloric acid, sodium acetate trihydrate, glacial acetic acid, aluminum chloride, sodium nitrate, sodium carbonate (anhydrous) and Celite *545 AW (Merck (Darmstadt, Germany), 2,2-diphenyl-1-picrylhydrazyl (DPPH), 2,2′-azinobis-3-ethylbenzothiazoline-6-sulfonic acid (ABTS), 6-hydroxy-2,5,7,8-tetramethylchroman-2-carboxylic acid (Trolox) and quercetin (Sigma-Aldrich, Steinheim, Germany), sodium hydroxide (Fisher Scientific, Loughborough, UK), sodium dihydrogen phosphate, disodium hydrogen phosphate and sodium acetate (Centrohem, Belgrade, Serbia), ethanol (Vrenje Spiritana, Belgrade, Serbia), acetone (J.T. Baker Chemical Co., Phillipsburg, NJ, USA) and Megazyme kit (Megazyme, Chicago, IL, USA) were used. All chemicals were of analytical grade.

#### 2.1.2. Apple Pomace Flour Production and Analysis

Whole apple pomace (AP) containing pulp, peel, seeds and stems, originated from mixed varieties of apples, with idared prevalent, grown conventionally in Serbian orchards in 2017, collected from the juice factory Fruvita (Smederovo, Serbia) immediately after pressing, was dehydrated at the industrial level for 5 h below 55 °C using “Solaris” dehydrator (Drayer ltd., Belgrade Serbia) [15]. Dried AP was ground into fine and coarse flour, packed in multilayer paper bags and kept in common storage conditions before analysis and application in confectionery. Water activity (a_w_) was determined by a_w_ meter Testo 650 (Testo AG, D-79853, Lenzkirch, Germany). Content of DF in APF was determined as described in Section 2.4.1. APF was extracted by occasional shaking with 1:1 mixture of ethanol and water (1400 μL) at room temperature during 60 min. Supernatant obtained by centrifugation at 12,000 rpm (Centrifuge MiniSpin® plus, Eppendorf, AG, Hamburg, Germany) for 10 min was tested for TPC, TFC and radical scavenging activity towards DPPH and ABTS radicals as described in Section 2.4.2 and Section 2.4.3.

### 2.2. Determination of Technological Properties of APF

Particle size analysis—particle size was determined by dry sieving after grinding. The particle size distribution of fine and coarse APF was determined by sieving 100 g of flour on a set of sieves with pore sizes of 1000, 500, 300, 160, 125, 90, 63 and 45 µm. The remaining mass on top of each sieve was weighed and used to calculate particle size distribution [26].

Bulk and packed density—a 50 mL graduated cylinder was filled with 10.0 g of fine or coarse APF. APF volumes were recorded for the level reached after gentle tapping (bulk density) or after applying pressure manually to achieve a maximal reduction of the volume (packed density). Results are expressed as weight per volume of APF (g/L) [27].

Water holding capacity (WHC)—APF samples of different particle size (fine and coarse; 1.0 g) were dissolved in distilled water (30 mL). The suspension of APF in distilled water (30 mL) was left for 24 h at room temperature. After centrifugation at 3600 rpm for 20 min, aqueous supernatant was removed and the residue was measured. Results are expressed as weight of water bound per gram of APF (g/g) [28].

Solubility—the aqueous supernatant that was removed after centrifugation during WHC determination was dried in the air oven at 105 °C until constant weight for subsequent analysis of solubility pattern. Water solubility index was determined from the quantity of dry solids obtained after drying the supernatant and is expressed as a percentage (%).

Hydrated density (HD)—APF (1.0 g) was added to a graduated cylinder filled with distilled water (10 mL) carefully, avoiding adhesion of particles to the cylinder walls. The difference between volume of water before and after APF addition was recorded after 15 min. Results are expressed as weight of APF (g) per water displaced (mL) [28].

Swelling capacity (SWC)—APF (1.0 g) was loaded into a graduated tube and 30.0 mL of water were added. After 18 h of equilibration, the bed volume was recorded. Results are expressed as volume occupied by APF (mL) per original APF weight (g) [29].

Oil holding capacity (OHC)—the mixture of APF (1.0 g) and 10.0 mL sunflower oil was kept for 24 h at room temperature without disturbance. After centrifugation at 3600 rpm for 25 min, supernatant was removed and weight of the residue measured. Results are expressed as gram of oil retained per gram of sample [27].

### 2.3. Production of Fortified Cookies with 25%, 50% and 75% of APF

Standard cookie dough (control) was prepared according to a traditional method. Ingredients acquired from the local suppliers (white wheat flour 59%, palm fat 19%, powdered sugar 14%, invert sugar 4%, salt 2% and baking powder 2%) were mixed in a single phase procedure. Fortified cookies were produced by replacing 25%, 50% and 75% of wheat flour with fine (F) and coarse (C) APF. They were marked as CF25, CF50, CF75 (CF-cookies with fine flour), CC25, CC50 and CC75 (CC-cookies with coarse flour). After passing through a two roll dough feed unit, cookies with a 40 mm diameter and 5–6 mm thickness were formed using a wire cut machine (Polin, Multidrop, Italy) and baked in an industrial electric deck oven at 175 °C for 10 min. Immediately after cooling cookies were packed in duplex polypropylene (PP) bags designed for food storage (BLIKPRODUKT" ltd. Kikinda, Serbia), closed and stored at ambient temperature. 

### 2.4. Determination of Fortified Cookies Functionality

#### 2.4.1. Determination of Dietary Fiber Content

Content of dietary fibers was determined in the APF and cookies of coarse flour, according to the AOAC met.985.29-enzymatic gravimetric method [30].

#### 2.4.2. Determination of Total Polyphenolics (TPCs) and Flavonoids Content (TFCs)

Finely ground cookies (100 mg) were extracted by occasional shaking with 1:1 mixture of ethanol and water (1400 μL) at room temperature during 60 min. Supernatant was obtained by centrifugation at 12,000 rpm for 10 min. TPC, TFC and AO activity (DPPH and ABTS) of extracts was determined immediately after extraction.

TPC of extracts was determined according to the procedure reported by Singleton and Rossi [31]. An aliquot of diluted extracts (0.25 mL) was mixed with Folin-Ciocalteu’s phenol reagent at 10-fold dilution (1.25 mL) and allowed to react for 6 min. Sodium carbonate solution (75 g/L, 1 mL) was added, and the mixture and shaken. After reacting for 2 h at room temperature in dark, absorbance was measured at 765 nm. The results are expressed as mg of gallic acid equivalents per gram of sample, mg (GAE)/g.

TFC of extracts was determined according to the procedure reported by Zhishen et al. [32]. An aliquot of extracts (2.5 mL) was mixed with 150 μL of a 5% NaNO_2_ solution and allowed to react for 6 min. 10% AlCl_3_ (150 μL) was added and the mixture was left to react for 5 min. Afterwards, 1 mL of 1 mol/L NaOH solution and 1.2 mL of distilled water were added and absorbance was measured at 510 nm. The results were expressed as mg of quercetin equivalents per gram of sample (QE)/g.

#### 2.4.3. Determination of Antioxidant Activity

The DPPH scavenging ability of extracts was determined according to a slight modification of the procedure reported by Kaneda et al. [33]. An aliquot of the diluted sample (0.2 mL) was mixed with 2.8 mL of DPPH solution (mixture of 1.86 × 10^−4^ mol L^−1^ DPPH in ethanol and 0.1 M acetate buffer (pH = 4.3) in volume ratio 2:1(*v*/*v*)). Free radical scavenging activity was determined by measuring the absorbance of the solution at 525 nm after 40 min of reaction at room temperature in the dark.

The ABTS scavenging ability of extracts was determined by the procedure reported by Re et al. [34]. By the reaction between 7 mM water solution of ABTS and 2.45 mM potassium persulfate (1:1), ABTS^+^ cation radical was produced. The obtained solution was stored in the dark at room temperature for 12–16 h before use. Thirty μL of each diluted sample was mixed with 3.0 mL of ABTS solution. After reacting for 6 min, absorbance at 734 nm was read. The results are expressed as mmol of Trolox equivalents per gram of sample (mmol TE/g).

### 2.5. Sensory Analysis of Fortified Cookies using a Scoring Method

Sensory quality rating was conducted in a laboratory for sensory evaluation in two testing periods: within two weeks after production (0 m) and after one year (12 m) of storage. The overall sensory quality was determined using the scoring method (0–5) whereby the representative sensory properties were evaluated: appearance (color, surface, size and shape), texture (structure, snap, doneness and chewiness) and flavor (odor and taste). Eight trained evaluators were engaged in the panel and pre-trained in two sessions. Multiplying the scores assigned by the panel—by the corresponding coefficients of importance (so called “weighted coefficient”), the weighted mean value of the scores was obtained as an expression of the final quality of the product. Quality categories were determined depending on score ranges: ˂2.5—does not meet the quality requirements; 2.5–3.5—good quality, 3.5–4.5—very good quality and 4.5–5.0—excellent quality.

### 2.6. Estimation of Fortified Cookies Storability

Having spent 12 months packed in plastic bags, stored in a cool and dark place at ambient temperature, the cookies were analyzed in the same way as described in Section 2.4. and Section 2.5.

### 2.7. Estimation of Optimal Share and Particle Size of APF

Principal components analysis (PCA) was conducted and changes in functional and sensorial properties during storage were considered in evaluation of optimal percentage of APF ratio and particle size.

### 2.8. Estimation of Consumer Acceptance of Fortified Cookies with Optimal APF Share

In accordance with the results of sensory quality judging, for the purpose of acceptance testing, a cookie sample containing 50% of coarse granulation APF was prepared. Consumer testing was performed by 115 students from the University of Belgrade. The chosen sample was evaluated for ‘overall acceptance’, ‘texture acceptance’, ‘flavor acceptance’ and ‘odor acceptance’ using the 9-point hedonic scale (1 = dislike extremely, 5 = neither like nor dislike and 9 = like extremely), and also, using 9-points just-about-right (JAR) scales (1 = too little, 5 = JAR and 9 = too much) for intensity of ‘color’ (too light/pale–JAR–too dark), ‘sweetness’ (not sweet enough–JAR–too sweet), ‘apple aroma’ (too weak–JAR–too strong), ‘crispiness’ (not crispy enough–JAR–too crispy), ‘doneness’ (underdone–JAR–overdone) and ‘chewiness’ (too easy to chew–JAR–too hard to chew).

### 2.9. Statistical Analysis

All experiments were performed in triplicate and the obtained results are expressed as mean ± standard deviation (SD). Data were subjected to analysis of variance (ANOVA) for comparison of means, and significant differences between groups were calculated according to Tukey’s HSD (honestly significant difference) test (*p* < 0.05). In order to determine the degree of change in antioxidative and sensory characteristics of the samples during the 12-month period, the PCA, followed by a Varimax rotation was performed. Statistical analyses were performed with XLSTAT (version 2014.5.03, Addinsoft, New York, NY, USA), analysis and statistics add-in for MS Excel.

Consumer acceptance data were subjected to mean drop analysis as described by Schraidt [35]. JAR scores were grouped into three categories: ‘below JAR’ (the scores 1, 2 and 3); ‘at JAR’ (4, 5 and 6) and ‘above JAR’ (7, 8 and 9). Mean drops were calculated by subtracting the mean overall hedonic score of each non-JAR category from the mean of the JAR category. The JAR-categories overall hedonic means were compared by ANOVA and Tukey’s HSD test. Minimum percentage skew for ‘Not Just Right’ (the cut-off) was set at 20% of the total consumer panel.

## 3. Results and Discussion

### 3.1. Production and Analysis of APF

All-natural gluten-free apple pomace flour (APF) without preservatives, artificial colors, dyes or any additives was produced within the scope of this study at industrial scale from whole apple pomace (AP; peel, pulp, stems and seeds), by dehydration followed by grinding to the desired particle size as disclosed [16]. It possesses low a_w_ (0.22), high DF content (42%), TPC (7.0 ± 0.4 mg GAE/g) and TFC (25.0 ± 1.5 mg QE/g) and prominent scavenging activity against DPPH and ABTS radical (9.4 ± 0.9 and 4.3 ± 0.3 mmol TE 100/g).

### 3.2. Technological Properties of Industrially Produced APF

Technological properties of fine and coarse APF were assessed (Table 1). Since APF was produced with the intention to be used in bakery and confectionery products, some of the parameters determined were compared with literature data available for wheat flour as well as commonly used gluten-free flours.

Average particle size of 0.16 and 0.5 mm for fine and coarse APF was determined by sieving, in the intervals of 0.06 to 0.30 and 0.06 to 1 mm, respectively. The maximum weight percentage for coarse flour is between 500 μm < *n* < 1 mm (28.32%), while for fine flour the distribution is slightly different and the maximum weight percentage is 27.78% for particle size between 160 μm < *n* < 300 μm.

Bulk density of fine and coarse APF was 435 ± 16 and 459 ± 20 g/L, and packed density 632 ± 25 and 591 ± 24 g/L, respectively. Bulk and packed density of sugar-depleted AP powder dominantly consisting of particles smaller than 150 μm was 557 and 447 g/L respectively [36]. Lowering of moisture content decreased the bulk density of apple powders obtained using various drying methods. The same trend was observed for tapped density of AP that ranged from 430 to 580 g/L [37]. Based on data available for other flours, it can be seen that while packed density values of APF are in concordance to those found in wheat flour (640 g/L) [38], bulk density is lower compared to other flours including wheat [39,40], rice [41] and corn [42]. According to Oladapo et al. [43], low values of bulk densities make the flour more suitable for the baking process.

Water holding capacity, referring to the ability of material to bind and hold water within the matrix, depends on the content and chemical structure of DF [44]. Reported AP WHC ranged from 1.62 g/g [45] to 6.34 g/g [46]. Values obtained for WHC of fine and coarse APF (4.69 ± 0.19 and 4.79 ± 0.18 g/g) were in concordance with previously reported data [36,47,48]. The main factors affecting WHC are related to the composition of AP, possible pre-treatment such as washing or bleaching, and drying conditions. Breakdown of cell wall polysaccharides does not occur at low dehydration temperatures, allowing for a high WHC of APF.

Since the amount of water needed to hydrate flour components to produce dough with optimum consistency is one of the most fundamental quality parameters of flour [49], values obtained for APF were compared with reported data for both wheat and other flours. The WHC values obtained for APF were found higher than those reported for fine (1.14 g/g) [50], full fat (1.85 g/g) and defatted (1.92 g/g) wheat flour [39], as well as commercial (0.88 g/g) [41], full fat (1.26 g/g) and defatted (1.56 g/g) rice [39] and corn flour (1.57 g/g) [51]. WHC of APF was also found higher than the WHC reported for oat bran (2.1 g/g) [52]. It is known that flours with a high WHC are widely used in foods like meat products, custards and soups to enhance thickening and viscosity, and in baked products to improve freshness and handling characteristics [10].

The ability of a powder to dissolve in water indicates its capacity of rehydration. Solubility affects the functional characteristics of powders in food systems. Lower values for fine and coarse APF solubility (27.9% ± 0.9% and 29.1% ± 0.7%) corresponded to high content of DF. Apple pomace powder solubility of 37.5% (total fiber content 26.5%) obtained by flash blanching, freeze-drying was reported [53].

High hydrated density of fine and coarse APF (0.50 ± 0.02 and 0.63 ± 0.03 g/mL) in comparison to reported data [28] can also be attributed to conditions of water removal that did not cause cell wall material shrinkage.

Swelling capacity, as well as WHC and OHC, provide insight regarding DF behavior during food processing and gut transit [54]. SWC, referred to the amount of insoluble fiber, was 5.5 ± 0.2 and 7.0 ± 0.3 mL/g for fine and coarse APF, respectively. SWC of sugar-depleted AP powder was found to be 7.0 mL/g [55]. Porous structures developed within the cell wall matrix during the process of water removal at low temperature allow for easy and complete rehydration.

Oil holding capacity of 1.27 ± 0.04 and 1.40 ± 0.05 g/g ascribed to fine and coarse APF, respectively, are in accordance with values reported for laboratory produced AP powder [47] and other plant raw materials [39]. Higher values were reported for boiled (1.69 g/g) [48,56] and washed AP (2.24 g/g) [55]. On the other hand, lower values were found in different rice flours, full fat (0.75 g/g) and defatted (1.1 g/g) [39], dry-milled (0.8 g/g), wet-milled (0.58 g/g) and commercial (0.5 g/g) [41]. OHC is related to the presence of lignin, its structure and surface properties, overall charge density, thickness, hydrophobic nature and size of particles [45,57]. Increase in drying temperature decreases the OHC value.

All values determined decreased with the decrease in APF particle size. Distinct effect of particle size on hydration capacity, oil absorption and emulsifying properties is in accordance with reported data [58]. Grinding may affect the hydration properties of DF as a result of an increase in surface area, leading to faster hydration [59]. However, it may cause alternation and collapse of the fiber matrix that traps water resulting in a water retention decrease. Both AP dehydration and dry AP grinding to fine and coarse APF in our study was obviously performed with no impact on the hydration characteristics of wet AP. Technological properties of APF confirmed its effectiveness in fortifying and development of DF-rich food products as well as in low calorie food.

### 3.3. Production of Fortified Cookies with 25%, 50% and 75% of APF

In order to demonstrate APF applicability in confectionery products fortification, an attempt was made to add more value to standard cookies, existing on the market for decades, by incorporating up to 75% of APF instead of wheat flour. Cookies were produced at a small-scale industrial level replacing 25%, 50% and 75% of soft wheat flour with both fine and coarse APF. Thermal behavior of APF was reported in our previous work. Thermal stability at up to 220 °C was demonstrated [17]. Temperature of cookies baking (175 °C) was much lower.

### 3.4. Functionality of Fortified Cookies

Level of fortification of the cookies i.e., functional characteristics of fortified cookies was determined based on an increase of phytochemicals content and AO activity. Significant improvement of standard cookies functionality, achieved by delivering both fiber and AOs through the incorporation of APF, was confirmed.

#### 3.4.1. Content of Dietary Fibers

Functional health-promoting cookies containing 10.1 ± 0.9, 20.6 ± 1.7 and 31.4 ± 2.9 g DF per 100 g were produced when replacing wheat flour with 25%, 50% and 75% APF, respectively while standard cookies with wheat flour only contained 1.7 ± 0.3 g DF per 100 g. It can be concluded that the cookies produced meet the requirements for high fiber foods [60].

#### 3.4.2. Content of Dietary Polyphenolics (TPCs) and Flavonoids (TFCs)

**Figure 1:**
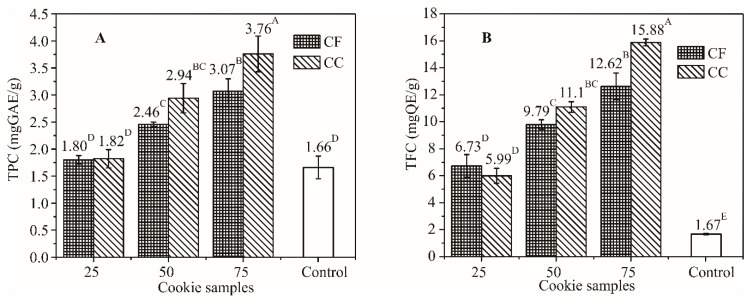
Increase of the total polyphenolic (TPC; **A**) and flavonoid content (TFC; **B**) of cookies in relation to the percentage of wheat flour substituted with coarse and fine APF (different superscripts indicate significant differences of means, according to Tukey’s HSD (honestly significant difference) test (*p* < 0.05)).

TPC and TFC, determined after production, corresponded to the percentage of APF added. Compared to the control sample, TPC was 1.1, 1.8 and 2.3 and 1.1, 1.5 and 1.9 times higher for cookies with 25%, 50% and 75% of coarse and fine APF, while TFC was increased 3.6-, 6.7- and 9.5- and 4.0-, 5.9- and 7.6-fold, respectively (Figure 1).

#### 3.4.3. Antioxidant Activity

**Figure 2:**
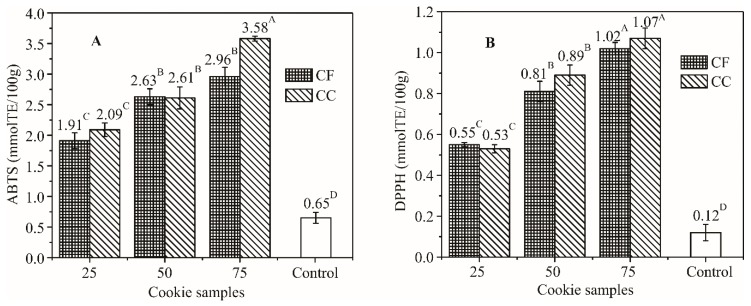
Increase of antioxidant (AO) activity of cookies determined by the ABTS (**A**) and DPPH (**B**) test in relation to the percentage of wheat flour substituted with coarse and fine APF (different superscripts indicate significant differences of means, according to Tukey’s HSD test (*p* < 0.05)).

Radical scavenging activity towards artificial radical species DPPH and ABTS increased 3.2-, 4.0- and 5.5- and 2.9-, 4.0- and 4.5-fold, and 4.4-, 7.4- and 8.9- and 4.6-, 6.7- and 8.5-fold, respectively (Figure 2).

The obtained results indicated that the substitution of wheat flour with APF significantly upgraded the content of DF, polyphenolics and flavonoids and the AO activity. At the same time, gluten content was significantly decreased. It is worth noting that all parameters measured were generally higher for cookies with coarse flour. Larger particles probably enable better preservation due to lower exposure of polyphenolics to the matrix.

Previous studies aimed at determining the effect of the addition of fruit and vegetable pomace in confectionery and bakery products also showed a significant increase in AO activity [61,62]. A significant progressive increase in TPC and TFC with the increase of AP level also was reported, along with an increase in radical scavenging and reducing ability [22]. Cakes prepared with AP were reported to have higher DF and polyphenolics contents than the control product, while the antioxidant property of AP was considered to function as a natural substitute for synthetic antioxidants [63]. In the recent study conducted by Alongi et al. [47], it was shown that partial replacement of wheat flour in short dough biscuits with 10% and 20% of AP led to a significant reduction of glycemic index, thus representing a possible strategy for diabetes type 2 management. Cookies with high APF share could be an even more promising final product in respect to glycemic index decrease.

### 3.5. Sensorial Properties of Fortified Cookies

In several researches, bakery and confectionery products various shares of AP incorporated were assessed for sensory properties and consumer acceptance. While products with the AP share of up to 20% usually had a pleasant apple flavor and good acceptance at the consumer level [19,50]—the sensory parameters more often decreased with a further increase in the AP ratio. Lower or moderate acceptability of cookies with 30%, 40% and 50% of AP was reported [24,25]. In concordance with previously published studies, cookies produced within the scope of this study with 25% of APF were best rated. From the obtained results (Table 2), it can be seen that in terms of overall sensory quality, the best rated cookies were CC25. They received the highest scores for all tested attributes. They were distinguished by typical appearance, corresponding texture and distinctive aroma. However, CC50 were also in the category of ‘excellent quality’ while all other samples had ‘very good quality’, including the control. Due mostly to an insufficiently baked layer in the structure of the cookies and a lack of crunchiness, CC75 received the lowest scores.

The use of laboratory produced AP powder increased the ratings for fruit and baking flavor of short dough biscuits, according to Alongi et al. [47]. While finer particle size of AP performed better than larger ones when used for cakes [63], this study revealed that cookies containing coarse APF possess better sensory properties along with superior nutritional value.

No differences in panel scores were observed previously for cookies with 30%, 40% and 50% of AP [25]. They all had lower scores in comparison to cookies produced and tested within the scope of this study. The average score for appearance was 3.1 ± 1.1; flavor, 3.0 ± 1.0, texture, 3.1 ± 1.1 and overall acceptability, 3.0 ± 1.0 (8-point hedonic scale of 1 = like extremely and 8 = dislike extremely was used) [25].

### 3.6. Storability of Fortified Cookies

#### 3.6.1. The Effect of Storage on the Functional Properties of Fortified Cookies

**Figure 3:**
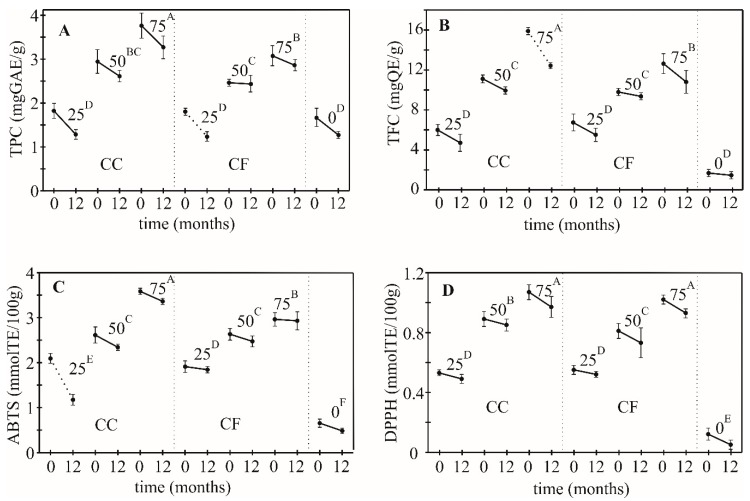
Decrease of TPC (**A**), TFC (**B**), scavenging of ABTS (**C**) and DPPH (**D**) test during 12 months storage of cookies (CC—cookies with coarse APF, CF—cookies with fine APF) in relation to the percentage of wheat flour substituted with APF (25%, 50% and 75%; different superscripts indicate significant differences of means, according to Tukey’s HSD test (*p* < 0.05), dashed line indicates the significant drop in value after 12 months (*p* < 0.05)).

After 12 months (12 m) no statistically significant change in the content of DF was observed. The differences among fortified and standard cookies (control) in terms of polyphenolics and flavonoids content and AO activity determined by ABTS and DPPH tests were found to be greater, but with the same trend (Figure 3).

Fortified cookies still have much higher values than control cookies. A decrease in the TPC value observed after storage was found significant only in the CF25 (*p* < 0.05). Values for TFCs significantly decreased in CC75 (*p* < 0.05). The DPPH values in all cookies remained unchanged after one year, while ABTS values decreased significantly only in CC25 (*p* < 0.05). Generally, the decrease was the lowest in cookies with 50% share of APF. The drop of TFC, TPC, ABTS and DPPH of CC50 (4.5%, 1.2%, 6.1% and 9.9%) and CF50 (10.5%, 11.2%, 10.3% and 4.5%) was significantly lower than in control (12.6%, 23.5%, 26.2% and 58.3%).

#### 3.6.2. The Effect of Storage on the Sensorial Properties of Fortified Cookies

With the aim to get an insight into changes in sensorial properties of cookies, the cookies were also assessed after 12 months, using the same method. After one year of storage, a slight decline in scores was noticed, but all samples remained in the initial quality category. The decrease of grades was due to expected modifications during long storage, such as color changes or loss of aroma and crunchiness, and not by the addition of APF (Table 3). Higher overall sensorial quality of CC25 and CC50 compared to the control in both testing periods indicates that there is a justification of the production of this type of functional cookies and that they have good prospects among consumers. In previous studies, AP powder used to fortify cookies also showed a positive influence on the cookies’ sensory attributes during storage. Cookies with 10% of AP maintained better sensory attributes and better storage stability compared to control cookies [36]. Results presented here support the hypothesis presented by Sahni and Shere [36] that fortified cookies might demand comparatively simple packaging.

The lowest changes of both sensorial and nutritional properties that occur in CC50 during storage favor the ratio of 50% of coarse flour as optimal for industrial production of fortified cookies.

The cookies containing coarse APF showed better sensory properties than those with fine APF, both at the beginning and at the end of the storage period. Although CC25 received a better score immediately after production, the decrease in AO activity and polyphenolic content during storage was the lowest for CC50. In this sense, CC50, as the choice superior to CC25 in terms of phytochemicals content and AO activity preservation during storage, could also be considered for consumer preferences testing using the hedonic scale.

### 3.7. Estimation of the Optimal Share and Particle Size of APF

**Figure 4:**
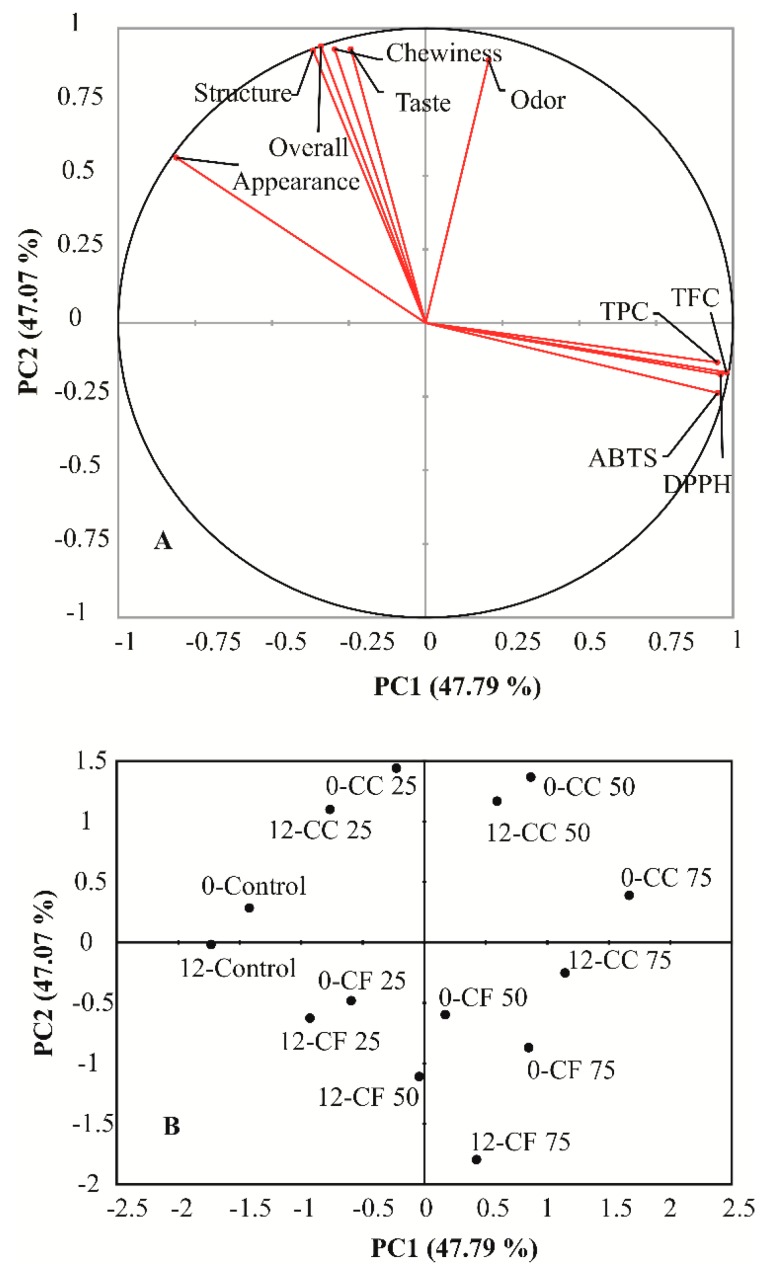
Principal component analysis (PCA) followed by Varimax rotation of samples (PC1 vs. PC2 plane): (**A**) correlation circle of variables and (**B)** samples in factor plane (samples at the beginning—prefix “0-“ and after 12 months—prefix “12-“).

To obtain a more comprehensive comparison between the produced cookies and to achieve as reliable a recommendation as possible regarding which ratio of APF to use in cookie fortification, functional and sensory characteristics were subjected to multivariate analysis by principal component analysis (PCA) followed by a Varimax rotation. Therefore, the following variables: TPC, TFC, ABTS, DPPH, appearance, structure, chewiness, odor, taste and overall quality were considered. The results showed that the first two principal components (PC) explain 94.86% of the total variance of initial data (PC1 explains 47.79%, and PC2 explains 47.07%). Projection of the initial variables in the PC1 vs. PC2 plane is shown in Figure 4A.

It can be seen that the PC1 is strongly related to variables associated with polyphenolic (TPC) and flavonoid (TFC) content and scavenging capacity (ABTS and DPPH). In the case of PC2, the most important variables are related to the sensory perception: structure, chewiness, odor, taste and overall quality. Thus, one can interpret the two first components, as follows: PC1—physicochemical characteristics (antioxidative property) and PC2—sensory characteristics of samples.

Samples at the beginning (prefix ”0-“) and after 12 months (prefix “12-“) in the PC1 vs. PC2 plane are shown in Figure 4B. The samples that are close to each other have similar properties, overall, and samples that are far apart are very different. To examine the influence of storage time on the change in functional and sensory characteristics of the samples, the distance between the points representing the sample at the beginning and after 12 months were observed. A larger distance indicates more intensive changes in the analyzed characteristics of sample over the observed time period. It can be seen that CC50 and CF25 changed the least with time in terms of functional and sensory properties. PCA results unequivocally confirm the better performance of coarse APF in cookie formulations. It allows CC50 to be reliably recommended for further production. Hence, consumers’ acceptance of cookies with 50% of coarse flour was further tested by hedonic tests.

### 3.8. Consumer Acceptability of Cookies with 50% of Coarse APF

**Figure 5:**
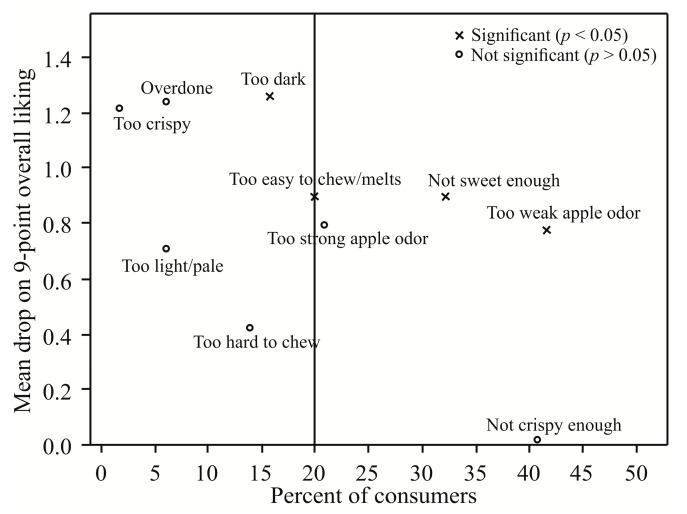
Mean drop analysis for the cookies with 50% of coarse APF (*n* = 115 respondents).

The results of the mean drop analysis for CC50 are shown in Figure 5. A point in the plot that shows a statistically significant mean drop and the percentage of consumers above the cut-off point is a cause for concern and suggests that the product be modified in the appropriate direction [64]. Two sufficiently large consumer groups (≥20%) with significant mean drops (*p* < 0.05) were identified for the tested product. One felt the product was ‘not sweet enough’ (32.2% of respondents), and the other that product had ‘too weak apple odor’ (41.7%). Average hedonic scores for the latter group were 4.3 ± 1.9 for ‘odor acceptance’ and 5.9 ± 1.5 for ‘overall acceptance’, i.e., more or less within the range of ‘neither like nor dislike’ category. The ‘apple-odor’ feature could be enhanced and intensified by proper selection of apple cultivars to be used in the production of this kind of product [65]. This property can also be affected by the degree of ripeness of the used fruits [66].

Looking at the total number of respondents, ‘overall acceptance’, ‘texture acceptance’ and ‘flavor acceptance’ were scored with the average hedonic scores above 6 (6.2 ± 1.8), indicating that the tested respondents liked the product.

## 4. Conclusions

This study showed that APF can be recommended as a flour fortifier and applied as an ingredient of functional products. Applicability of APF in industrial production of fortified cookies was demonstrated. Coarse APF performed better, both regarding functional properties and sensorial attributes in comparison to fine APF. Cookies fortified with coarse APF possessed a higher content of health promoting components in comparison to control samples, while its decrease during storage was lower. According to the scoring method, APF can be incorporated up to the level of 50% in cookie recipes, without significantly affecting sensorial properties. Consumers’ evaluation using a hedonic test also confirmed that replacement of up to 50% of wheat flour with coarse APF at industrial-scale production of cookies was possible, without compromising products acceptability. APF’s potential in bridging DF and AOs gaps, while also conferring a pleasant apple taste and crunchy texture that lasted throughout 12 months storage, was shown.

## 5. Patents

Zlatanović, S., Gorjanović, S., Ostojić, S., Micić, D., Pastor, F., Kalušević, A. and Laličić-Petronijević, J. PCT/RS2019/000019, 2019. International Patent Application.

## Figures and Tables

**Table 1:** Technological properties of coarse and fine apple pomace flour (APF).

Technological Properties	APF (Fine)	APF (Coarse)
Bulked density (g/L)	435 ± 16	459 ± 20
Packed density(g/L)	632 ± 25	591 ± 24
Water holding capacity (g/g)	4.69 ± 0.19	4.79 ± 0.18
Solubility (%)	27.9 ± 0.9	29.1 ± 0.7
Hydrated density (g/mL)	0.50 ± 0.02	0.63 ± 0.03
Swelling capacity (mL/g)	5.5 ± 0.2	7.0 ± 0.3
Oil holding capacity (g/g)	1.27 ± 0.04	1.4 ± 0.05

**Table 2:** Sensory evaluation of cookies with 25%, 50% and 75% of fine and coarse APF after production.

	Appearance	Structure, Snap, Doneness	Chewiness	Odor	Taste	Overall
Control	4.81 ± 0.12 ^a^	4.44 ± 0.12 ^ab^	4.16 ± 0.23 ^bcd^	4.66 ± 0.44 ^abc^	4.34 ± 0.13 ^abc^	4.46 ± 0.10 ^b^
0-CF25	4.06 ± 0.12 ^cd^	3.94 ± 0.18 ^cde^	4.06 ± 0.12 ^cd^	4.12 ± 0.23 ^cd^	4.25 ± 0.19 ^bc^	4.10 ± 0.08 ^cd^
0-CF50	3.84 ± 0.13 ^cde^	3.81 ± 0.26 ^de^	3.75 ± 0.33 ^def^	4.19 ± 0.22 ^cd^	4.06 ± 0.12 ^cd^	3.94 ± 0.10 ^de^
0-CF75	3.59 ± 0.13 ^ef^	3.50 ± 0.23 ^ef^	3.34 ± 0.13 ^ef^	4.44 ± 0.32 ^abcd^	3.63 ± 0.44 ^de^	3.70 ± 0.21 ^ef^
0-CC25	4.59 ± 0.33 ^ab^	4.75 ± 0.00 ^a^	4.75 ± 0.35 ^a^	4.84 ± 0.13 ^a^	4.84 ± 0.19 ^a^	4.77 ± 0.11 ^a^
0-CC50	4.06 ± 0.18 ^cd^	4.66 ± 0.13 ^a^	4.50 ± 0.33 ^abc^	4.81 ± 0.12 ^a^	4.75 ± 0.19 ^ab^	4.59 ± 0.11 ^ab^
0-CC75	3.66 ± 0.30 ^def^	3.94 ± 0.18 ^cde^	3.84 ± 0.19 ^de^	4.75 ± 0.19 ^ab^	4.13 ± 0.40 ^cd^	4.09 ± 0.14 ^d^

The values are presented as mean ± SD, different superscripts within the same column indicate significant differences of means, according to Tukey’s HSD test (*p* < 0.05).

**Table 3:** Sensory evaluation of cookies with 25%, 50% and 75% of fine and coarse APF after 12 months of storage.

	Appearance	Structure, Snap, Doneness	Chewiness	Odor	Taste	Overall
12-control	4.81 ± 0.12 ^a^	4.41 ± 0.30 ^abc^	4.13 ± 0.23 ^bcd^	4.50 ± 0.40 ^abcd^	4.25 ± 0.30 ^bc^	4.39 ± 0.18 ^bc^
12-CF25	4.22 ± 0.21 ^bc^	4.03 ± 0.36 ^bcd^	3.94 ± 0.12 ^cd^	4.13 ± 0.27 ^cd^	4.13 ± 0.13 ^cd^	4.08 ± 0.12 ^d^
12-CF50	3.91 ± 0.19 ^cde^	3.72 ± 0.41 ^de^	3.34 ± 0.27 ^ef^	4.22 ± 0.21 ^bcd^	3.63 ± 0.30 ^de^	3.75 ± 0.18 ^e^
12-CF75	3.34 ± 0.30 ^f^	3.22 ± 0.39 ^f^	3.19 ± 0.32 ^f^	4.06 ± 0.44 ^d^	3.25 ± 0.52 ^e^	3.41 ± 0.26 ^f^
12-CC25	4.66 ± 0.30 ^a^	4.75 ± 0.19 ^a^	4.66 ± 0.44 ^ab^	4.81 ± 0.22 ^a^	4.59 ± 0.30 ^abc^	4.69 ± 0.10 ^ab^
12-CC50	4.09 ± 0.13 ^c^	4.50 ± 0.19 ^ab^	4.44 ± 0.55 ^abc^	4.81 ± 0.22 ^a^	4.72 ± 0.25 ^ab^	4.54 ± 0.17 ^ab^
12-CC75	3.50 ± 0.19 ^ef^	3.81 ± 0.26 ^de^	3.72 ± 0.21 ^def^	4.66 ± 0.13 ^abc^	3.69 ± 0.12 ^de^	3.88 ± 0.13 ^de^

The values are presented as mean ± SD, different superscripts within the same column indicate significant differences of means, according to Tukey’s HSD test (*p* < 0.05).
